# Intracellular Ca^2+ ^regulating proteins in vascular smooth muscle cells are altered with type 1 diabetes due to the direct effects of hyperglycemia

**DOI:** 10.1186/1475-2840-9-8

**Published:** 2010-02-01

**Authors:** Yvonne M Searls, Rajprasad Loganathan, Irina V Smirnova, Lisa Stehno-Bittel

**Affiliations:** 1 Department of Physical Therapy and Rehabilitation Science, University of Kansas Medical Center, Kansas City, KS 66160, USA

## Abstract

**Background:**

Diminished calcium (Ca^2+^) transients in response to physiological agonists have been reported in vascular smooth muscle cells (VSMCs) from diabetic animals. However, the mechanism responsible was unclear.

**Methodology/Principal Findings:**

VSMCs from autoimmune type 1 Diabetes Resistant Bio-Breeding (DR-BB) rats and streptozotocin-induced rats were examined for levels and distribution of inositol trisphosphate receptors (IP_3_R) and the SR Ca^2+ ^pumps (SERCA 2 and 3). Generally, a decrease in IP_3_R levels and dramatic increase in ryanodine receptor (RyR) levels were noted in the aortic samples from diabetic animals. Redistribution of the specific IP_3_R subtypes was dependent on the rat model. SERCA 2 was redistributed to a peri-nuclear pattern that was more prominent in the DR-BB diabetic rat aorta than the STZ diabetic rat. The free intracellular Ca^2+ ^in freshly dispersed VSMCs from control and diabetic animals was monitored using ratiometric Ca^2+ ^sensitive fluorophores viewed by confocal microscopy. In control VSMCs, basal fluorescence levels were significantly higher in the nucleus relative to the cytoplasm, while in diabetic VSMCs they were essentially the same. Vasopressin induced a predictable increase in free intracellular Ca^2+ ^in the VSMCs from control rats with a prolonged and significantly blunted response in the diabetic VSMCs. A slow rise in free intracellular Ca^2+ ^in response to thapsigargin, a specific blocker of SERCA was seen in the control VSMCs but was significantly delayed and prolonged in cells from diabetic rats. To determine whether the changes were due to the direct effects of hyperglycemica, experiments were repeated using cultured rat aortic smooth muscle cells (A7r5) grown in hyperglycemic and control conditions. In general, they demonstrated the same changes in protein levels and distribution as well as the blunted Ca^2+ ^responses to vasopressin and thapsigargin as noted in the cells from diabetic animals.

**Conclusions/Significance:**

This work demonstrates that the previously-reported reduced Ca^2+ ^signaling in VSMCs from diabetic animals is related to decreases and/or redistribution in the IP_3_R Ca^2+ ^channels and SERCA proteins. These changes can be duplicated in culture with high glucose levels.

## Background

While improved care for people with diabetes has reduced the overall risk of complications and mortality, rates of mortality for people with diabetes is still two fold higher than those without diabetes [1,2]. Cardiovascular disease accounts for a significant percentage of deaths of individuals inflicted with type 1 diabetes [3-7]. Even in young adults, cardiovascular events such as stroke are higher in people with type 1 diabetes than in those without diabetes [8]. As the duration of life with diabetes increases, so increases the risk of cardiovascular events and mortality [9]. Current management procedures focusing on tight glycemic control may have only marginal effects to halt the progression of large vessel disease [10].

While the majority of research effort concerning diabetic vascular disease has been focused on changes in the endothelial cells, alterations in the vascular smooth muscle cells (VSMCs) are also known to occur [11-13]. The VSMCs surrounding vessel walls, undergo increased proliferation, adhesion, and migration in conditions of hyperglycemia [14-17]. Several proteins and genes have been proposed as targets for hyperglycemic changes in the VSMCs including protein kinase C (PKC). Interestingly, many of them have a common upstream pathway that includes the release of calcium (Ca^2+^) from intracellular stores, including the sarcoplasmic reticulum (SR).

In VSMCs there are two channels that have been identified as important in the release of intracellular Ca^2+ ^stores; the inositol 1,4,5-trisphosphate (IP_3_)-sensitive store and the ryanodine-sensitive store [18]. In VSMCs, IP_3 _release of Ca^2+ ^from intracellular stores is a common pathway for activation of many proteins including PKC [19] and PLC. Changes in basal levels of intracellular Ca^2+ ^with diabetes are inconsistent, but Ca^2+ ^transients in response to agonists such as angiotensin II are clearly attenuated in VSMCs from diabetic animals [20]. The blunted IP_3_-induced Ca^2+ ^responses could be due to changes in the intracellular Ca^2+ ^channels, the intracellular Ca^2+ ^storage site (SR), or the sarcoplasmic/endoplasmic Ca^2+ ^ATPase (SERCA). This study examines changes in the intracellular Ca^2+ ^regulatory proteins in two animal models of type 1 diabetes. The streptozotocin-induced diabetic rat is a well-established rat model using a toxin to destroy the pancreatic beta cells. The second model utilizes the DR-BB rat, which better mimics the autoimmune component of type 1 diabetes. Results from these animal models were compared to a cell culture model (A7r5) with hyperglycemia.

## Materials and methods

### Induction of Diabetes

#### Streptozotocin Rat Model

Seven week old male Sprague Dawley rats (Harlan, Indianapolis, IN) weighing 250-270 grams were injected intraperitoneally with 65 mg streptozotocin (STZ, Sigma, St. Louis, MO) per kg body weight (n = 15). Non-diabetic control rats (n = 12) were injected with vehicle (citrate buffer). Blood glucose levels greater than 250 mg/dL, measured by an Accu-Check Advantage glucometer (Boehringer Mannheim Corporation, Indianapolis, IN), indicated the development of diabetes.

The blood glucose levels and body weights of all rats were assessed weekly. The final weight and blood glucose levels are shown in Table 1. Free access to food and water was supplied. The principles of institutional laboratory animal care were strictly followed. The rats were killed with an intraperitoneal injection of pentobarbital after 8 weeks of diabetes.

**Table 1:** Characteristics of Animal Models of Diabetes

	Physiological Parameters			
	**STZ-Induced Diabetic Rat Model**		**DR-BB Diabetic Rat Model**	

	**Control**	**Diabetic**	**Control**	**Diabetic**

Weight (g)	453 ± 16	328 ± 14 *	217 ± 5	212 ± 19

Blood Glucose (mg/dL)	113 ± 5	554 ± 13 *	98 ± 2	295 ± 13 *

*Indicates a significantly different value when compared to respective controls (p < 0.001)

#### DR-BB Rat Model

Diabetes was induced in 21-26 day old Diabetes Resistant Bio-Breeding (DR-BB) Wistar rats using anti-RT6 monoclonal antibody (Dr. Dale Greiner, University of Mass) DS4.23 hybridoma in 2 ml of tissue culture medium injected 5 times/week in combination with polyinosinic-polycytidylic acid (Sigma), a non-specific immune system activator at 5 μl/g of body weight 3 times/week. Induction treatment was discontinued at the onset of diabetes as determined by a blood glucose level above 250 mg/dL. Diabetes typically occurred 2-3 weeks after the treatment was initiated. Within 1 week of diabetes development, the diabetic rats were treated with insulin (Humulin R, Humulin U and Humulin N; Eli Lilly, Indianapolis, IN) through subcutaneous implantation of an insulin-filled Alzet osmotic pump (Alza, model 2004, 0.25 μl/hr for 28 days; Mountain View, CA). Blood glucose was closely monitored for the remaining time, and hyperglycemic animals were given insulin injections as needed in amounts based upon the severity of hyperglycemia. Control rats underwent the same manipulations except pumps were filled with saline. Body weight and blood glucose were followed weekly, with the final values shown in Table 1. Diabetic animals (n = 7 female, 4 male) and their age matched controls (n = 6 female, 4 male) were euthanized after 8 weeks of diabetes.

### Cell Dispersion

Dissected aortas and femoral arteries were cleaned of adventitia in cold phosphate buffered saline (PBS) buffer. The vessels were cut lengthwise to expose the intima, which was gently swabbed to remove endothelial cells. Each vessel was pinned, lumen side up, in a small covered beaker containing Sylard Gel (Dow Corning, Midland, MI) and bathed in collagenase solution containing deoxyribonuclease I, collagenase type 2, soybean trypsin inhibitor (Worthington Biochemical Corporation, Lakewood, NJ) and bovine albumin (Sigma). After 30 minutes of shaking at 37°C, the first digest was discarded as it contained predominantly endothelial cells. New collagenase solution was added and further dispersions were gathered every 2 hours. Cells were gently centrifuged to remove the enzyme solution before being rinsed and re-suspended in media.

### Cell Culture

Cells from the A7r5 cell line (American Type Tissue Culture, Manassas, VA), derived from rat thoracic aorta, were grown in Dulbecco's Modified Eagle's Medium supplemented with 10% fetal bovine serum, penicillin, and streptomycin at 37°C, 5% CO_2_. Cells were grown in flasks, then seeded in 100 mm dishes or on coverslips with glucose concentrations of either the supplier-recommended concentration (25 mM; referred to as medium) or high glucose (75 mM). Because 25 mM glucose is high compared to physiological serum levels, a third (low) glucose concentration was tested (5.5 mM). The effects of osmolarity were controlled by the addition of mannitol. Cells were grown to semi-confluence prior to testing.

### Immunoblotting

Total proteins were extracted from tissue or cell culture using lysis buffer containing 10 mM Tris, pH 7.4, 1 mM sodium ortho-vanadate and 1% SDS. To separate nuclear and cytoplasmic proteins, the NE-PER kit (Pierce, Rockford, IL) was used. Each sample was concentrated by ultrafiltration using Microcon YM-10 (Millipore, Bedford, MA). Protein concentrations were measured in 96-well format using the BCA kit (Pierce, Rockford, IL) and a MRX microplate reader (Dynex Technologies, Chantilly, VA). A total of 30 or 50 μg of protein was loaded in each lane of 8, 10 or 4-15% gels. Rat cardiac tissue extract was used as a positive control for ryanodine receptor (RyR) and A-10 cell lysate (Santa Cruz, Santa Cruz, CA) was used for Inositol 1,4,5 trisphosphate receptors (IP_3_Rs). After electrophoretic separation, proteins were transferred from a gel to a PVDF membrane (Pierce) overnight. Ponceau's stain (Sigma) was used to assess protein loading prior to blotting. A standard immunoblotting protocol was performed. Primary antibodies to SERCA2, SERCA3, IP_3_R type I and RyR (Affinity BioReagents, Golden, CO), and IP_3_R type 2 and IP3R type 3 (Santa Cruz) were used. GAPDH was used as a housekeeping gene product (antibody, Santa Cruz). Anti-HSP90 (Santa Cruz) was used to assess the purity of the cytoplasmic extractions and anti-p62 (BD Transduction, Lexington, KY) or anti-USF-2 (Santa Cruz) were used to test the purity of the nuclear extracts. Detection was performed using enhanced chemiluminescent reagent (Pierce) or SuperSignal West Pico Chemiluminescent reagent (Pierce). Duplicates were made of each immunoblot experiment.

### Immunocytochemistry

A7r5 cells were grown on cover slips in various glucose concentrations. Freshly dispersed VSMC were allowed to attach to glass coverslips for at least 2-4 hours in PBS at 37°C prior to fixation with 2% paraformaldehyde (Fisher Scientific, Palatine, IL). Fixed cells were rinsed with PBS, permeabilized with 1% Triton X-100 (Sigma) and rinsed well before blocking in 10% goat or donkey serum (Jackson Immunoresearch Laboratories, West Grove, PA). Cells were incubated with primary antibody (same as above) diluted in 5% non-fat dry milk (Bio-Rad, Helcules, CA) solution overnight in a cold box at manufacturer recommended concentrations. Alpha smooth muscle actin (Sigma) was used to identify SMC type. Cells were repeatedly rinsed in PBS before incubation with secondary antibody conjugated to Rhodamine Red or Cy 2 (Jackson ImmunoResearch) in a dark chamber. Brefeldin A (Invitrogen, Carlsbad, CA) was used to evaluate sarcoplasmic reticulum (SR). Coverslips were rinsed and allowed to air dry in the dark before mounting. Images were captured using an Olympus Fluoview 300 Confocal Microscope with the consistent confocal settings for each pair of control and diabetic cells per single primary antibody. Experiments were performed in triplicate.

### Live Cell Intracellular Calcium Measurements

Freshly dispersed VSMCs or cultured A7r5 cells were allowed to attach to coverslips for 1 hour in PBS at 37°C before loading with 1 μM each of two Ca^2+ ^sensitive fluorophores, Fluo-4/AM and Fura-Red/AM (invitrogen) by incubation at 37°C for 30-60 minutes. The coverslips were inserted into an Attofluor chamber (Molecular Probes) and rinsed with warm PBS. PBS with ethylene glycol tetraacetic acid (EGTA) to buffer extracellular Ca^2+ ^(0 Ca^2+^), was added to the chamber mounted on the stage of a confocal microscope so that the measured Ca^2+ ^changes were limited to the intracellular stores only. Baseline measurements were taken on quiescent cells for 3-4 minutes prior to the application of any agonist. Vasopressin (10 nM, Sigma) or thapsigargin (20 μM, Sigma) were added to the bath at the times indicated in the results section. Images were collected every 5 or 10 seconds using an inverted Nikon Eclipse TE 300/Bio-Rad MicroRadiance Plus Laser Scanning Confocal Microscope (Bio-Rad Laboratories, Germany) or an Olympus Fluoview 300 confocal microscope (Olympus, Center Valley, PA). Fluoview software was used to analyze Ca^2+ ^signaling experiments. Time-to-peak was defined as the time of agonist application to the time the maximal magnitude of the response was obtained. The duration of the Ca^2+ ^signal was defined as the time from the upstroke of the response to the return within 25% of initial baseline value.

### Data analysis

All data points were normalized for background signal prior to statistical analysis. Fluoview software (Olympus, Melville, NY) was used to analyze Ca^2+ ^signaling experiments. Adobe Photoshop (Adobe Systems, San Jose, CA) was used to analyze relative fluorescence intensity, and color analysis. Analysis of variance was performed for Ca^2+ ^fluorescence and immunoblot density values using Sigma Stat software. When appropriate, statistical differences were assessed using Dunnett's test for multiple comparisons after a one-way analysis of variance using Sigma Stat (SPSS Inc, Chicago, IL). A probability level of p < 0.05 was defined as a significant difference.

## Results

### Animal Models of Diabetes

The blood glucose levels were significantly higher (p < 0.001) in both diabetic rat models. However, the animals from the toxin-induced diabetes (STZ) were significantly more hyperglycemic when compared to the genetically-prone diabetic group (DR-BB) (Table 1, p < 0.001). This difference is due to the fact that uncontrolled diabetes is lethal in the DR-BB model and the animals were provided an insulin pump or injected with insulin daily after each became diabetic. In contrast, the STZ animals received no exogenous insulin. The body weight of control and diabetic DR-BB rats were comparable but in the STZ diabetic rat model, the diabetic rats demonstrated a significant decline in weight over time (Table 1, p < 0.001).

### Diabetes Attenuated the Ca^2+ ^responses in Freshly Dispersed SMCs

**Figure 1:**
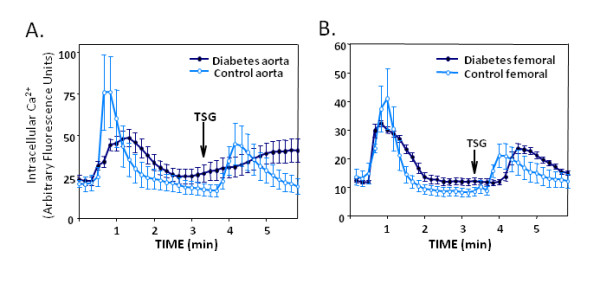
**Intracellular Ca^2+ ^Transients were Altered by Diabetes**. Whole cell vasopressin and thapsigargin-induced Ca^2+ ^transients were altered by diabetes in VSMC from the aorta and femoral artery. Vasopressin was applied to VSMCs from the aorta (A) or femoral artery (B) from DR-BB rats at time 0. The time of the thapsigargin application is shown with the arrow (TSG). In both cases the vasopressin-induced Ca^2+ ^transients were blunted in amplitude and thapsigargin responses were delayed in cells from diabetic rats. For all figures, blue shades indicate data of cells from diabetic and control rats; green shades indicate data from cultured A7r5 cells grown in various glucose concentrations.

Freshly dispersed VSMC from arteries of control and diabetic rats were evaluated for agonist-induced Ca^2+ ^transients. Whole cell Ca^2+ ^fluoremetry indicated that the responses to vasoactive substances were significantly blunted in the VSMC from diabetic animals. Mean Ca^2+ ^responses to vasopressin and thapsigargin were both blunted in VSMC from the aorta of DR-BB diabetic rats (Figure 1A). In cells taken from femoral arteries, the vasopressin response was blunted, but diabetes had no effect on the peak of the thapsigargin response (Figure 1B). The peak of the vasopressin responses were delayed only in the STZ diabetic animal model, and the thapsigargin responses were delayed in both animal models (Table 2). In addition, fewer VSMC from diabetic animals responded to the vasopressin stimulation. VSMC from control animals responded to vasopressin 78% of the time, while only 45% of the VSMCs from diabetic rats responded to vasopressin exposure. A decline in the vasopressin amplitude of the responding cells was also measured in VSMC from the STZ-induced diabetic animals when compared to the controls (results not shown).

**Table 2:** Delay in Agonist-Induced Ca^2+ ^Transient in SMC from Diabetic Animals

	Ca^2+ ^Response (Time to Peak in seconds)			
	STZ-Induced Diabetic Rat Model		DR-BB Diabetic Rat Model	
	Control	Diabetic	Control	Diabetic
Vasopressin	17 ± 3	54 ± 14 *	22 ± 2	26 ± 3
Thapsigargin	35 ± 3	67 ± 9 *	41 ± 7	152 ± 23 *

*Indicates a significantly different value when compared to respective controls (p < 0.05)

**Figure 2:**
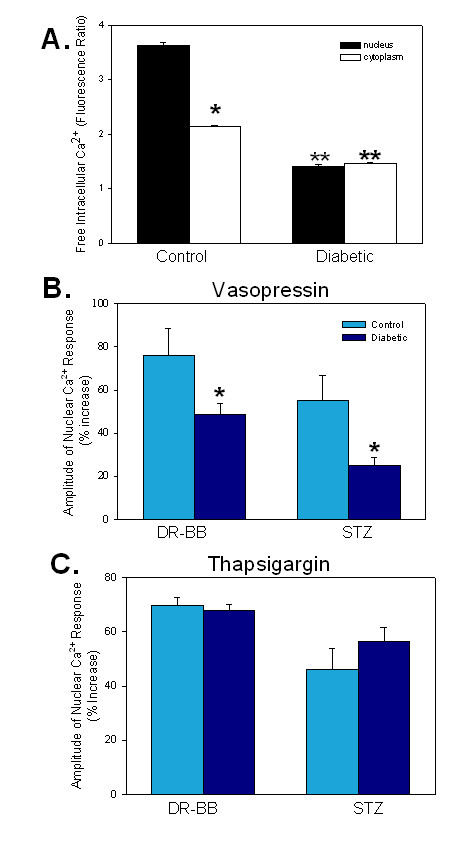
**Peak Ca^2+ ^Transients were Blunted in Diabetes**. A) Separate analysis of the nuclear and cytoplasmic compartments indicated that most of the diabetes-induced changes in the Ca^2+ ^transients occurred due to changes in the nuclear compartment. Ratiometric Ca^2+^-sensitive fluorophores demonstrated the decline in the resting Ca^2+ ^levels with diabetes that were especially dramatic in the nuclear compartment. The figure illustrates results of cells from DR-BB rats. P < 0.05. * indicates a significant difference in the fluorescence levels between the nuclear and cytoplasmic compartments in VSMC from control animals. ** indicates a significant decline in the resting Ca^2+ ^concentration in cells from diabetic animals compared to the control. There was no statistical difference noted in the nucleus and cytoplasm within cells from diabetic aorta. B) The amplitude of the vasopressin-induced *nuclear *Ca^2+ ^transients declined with diabetes in both diabetic animal models. * indicates a significant difference between the control and diabetic groups, p < 0.05. C) There was no significant change in the amplitude of the thapsigargin-induced *nuclear *Ca2+ transients in either diabetic animal model. * indicates p < 0.05. For Figure 2, n = 39 and 42 cells from control and diabetic STZ rats, respectively. n = 24 and 22 cells from control and diabetic DR-BB rats, respectively.

To further define the changes in the Ca^2+ ^signal, ratiometric fluorophores were utilized. With the ratiometric dyes, it was clear that the nuclear and cytoplasmic compartments responded differently to agonist stimulation. Resting Ca^2+ ^concentrations in the nucleus were higher than the cytoplasmic levels in control animals respective to STZ or DR-BB diabetic rats (p < < 0.001). With diabetes, the resting Ca^2+ ^concentration declined in both the nucleus and the cytoplasm; however, the nuclear decline was greater so that quiescent cells from diabetic animals had similar nuclear and cytoplasmic Ca^2+ ^concentrations (Figure 2A).

While whole-cell Ca^2+ ^peaks declined by 30% with diabetes (Figure 1), separate analysis of the cytoplasmic and nuclear compartments demonstrated that most of the difference was attributable to the nuclear compartment. Figure 2B illustrates a significant decline in the amplitude of the vasopressin-induced Ca^2+ ^peak in the nuclear compartment of both diabetic animal models compared to their controls as determined by the change of the fluorescence ratio (p < 0.01). The amplitude of the nuclear thapsigargin response was not different in VSMC from control or diabetic animals using either animal model (Figure 2C).

In order to rule out general changes in the nuclear envelope that could alter Ca^2+ ^signaling, two nucleus-specific proteins were analyzed. Both p62 and NUP153 are core components of the nuclear pore complex, and levels and distribution of both proteins has been shown to be associated with specific disease states [21]. Immunoblotting for p62 showed no difference in protein levels in samples from control or diabetic animals. In the STZ model of diabetes, the aortic VSMC p62 protein band density level was 67 ± 15 (arbitrary units) in control animals, and 70 ± 16 in diabetic tissue (no statistical difference). Similar results were found in the DR-BB aortic arteries. The values for p62 protein bands were 114 ± 6 in controls and 92 ± 15 in diabetes. In the femoral artery of the DR-BB animals, the levels were 62 ± 10 for controls and 77 ± 6 in diabetic rats. Like p62, levels of NUP153 also were not statistically different in the STZ or DR-BB rats in either the aortic or femoral VSMCs, suggesting that changes in nuclear Ca^2+ ^transients were not likely associated with gross changes in nuclear transport.

### Hyperglycemia attenuates Ca^2+ ^responses in cultured VSMCs

**Figure 3:**
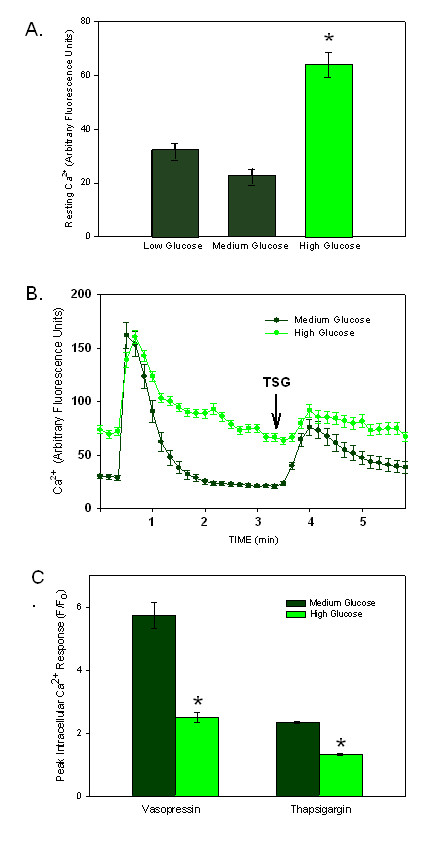
**Peak Ca^2+ ^Transients are Blunted in Response to Hyperglycemia in Culture**. A) Basal intracellular Ca^2+ ^concentrations increased in VSMCs when grown in high glucose conditions as opposed to low or medium glucose (n = 30 cells/group). * Indicates p < 0.0001. B) Stimulation of cells with vasopressin (time 0) and thapsigargin (TSG, arrow) illustrated blunted responses in cells grown in high glucose (n = 29 cells). C) The plot of the amplitude of the response to each agonist (normalized for the different basal Ca^2+ ^levels) illustrates the differences in the peak responses to vasopressin and thapsigargin (* indicates p < 0.05; n = 97 cells in low glucose and 138 in high glucose conditions).

To determine whether the blunted agonist responses were due directly to the exposure to hyperglycemia or were a physiological adaptation to long term diabetes, cultured rat aortic VSMCs were grown in high glucose. Figure 3A illustrates the increase in the resting intracellular Ca^2+ ^concentration that was 2-3 times higher in cells grown in high glucose, when compared to cells grown in low or medium (the supplier's suggested glucose concentration). These findings are opposite of those measured in cells from diabetic animals (either model) and may explain some of the confusion in the field regarding changes in resting cytoplasmic Ca^2+ ^concentrations with diabetes. There was a significant difference between the high glucose condition and the other two conditions (p < 0.0001). There was no statistically significant difference in the free intracellular Ca^2+ ^between the low glucose condition and the medium glucose condition. In fact, there was no statistical difference between the low and medium glucose conditions for all assays tested. Therefore, only medium and high glucose conditions are reported in this study.

When stimulated with vasopressin or thapsigargin, cells responded with an immediate increase in intracellular Ca^2+ ^that was diminished by high glucose conditions. Figure 3B illustrates the altered responses to both vasopressin (time 0) and thapsigargin (TSG, arrow) from cells grown in medium and high glucose conditions. The different resting Ca^2+ ^levels made it difficult to determine a change in vasopressin- induced peak responses. In order to compare the amplitude of the responses, the differences in baseline Ca^2+ ^concentrations were normalized by dividing values by the resting Ca^2+ ^fluorescence values (F/Fo) following the methods reported previously [22]. The normalization clearly illustrates the attenuated peak response of the cells to either agonist in hyperglycemic conditions (Figure 3C).

**Figure 4:**
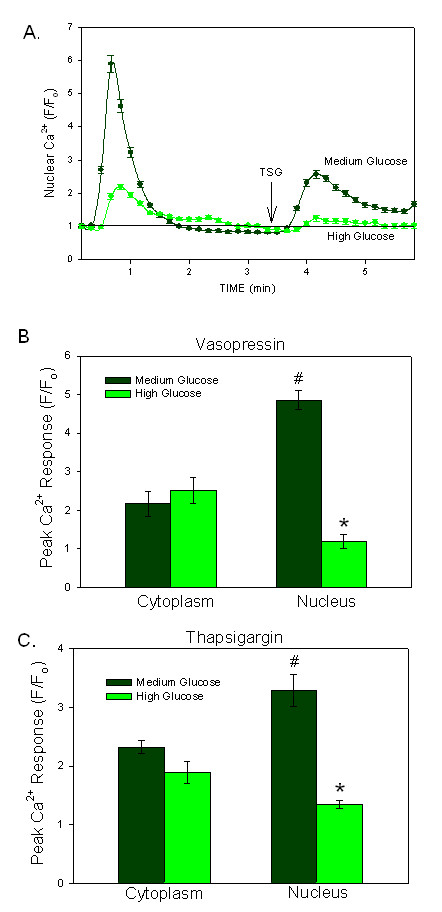
**Nuclear Ca^2+ ^Transients are Altered by High Glucose**. A) *Nuclear *Ca^2+ ^responses to vasopressin and thapsigargin (TSG) in A7r5 cells grown in medium glucose or high glucose conditions are shown. Both the vasopressin (added at time 0) and the TSG (added at the arrow) responses were blunted in the nuclei of cells grown in high glucose. (n = 15 cells/group, p < 0.0001) B) The mean peak amplitude of the Ca^2+ ^response to vasopressin did not change significantly in the cytoplasm when cells were grown in high glucose. However, the nuclear compartment showed significant reduction of the vasopressin response in high glucose (n = 94 cells) versus medium glucose (n = 37). (p < 0.001; indicated by *) C) The mean peak amplitude of the thapsigargin (TSG) response did not change significantly in the cytoplasmic compartment with high glucose exposure, but was significantly blunted in the nuclear compartment (n = 37). (p < 0.001; indicated by *).

As noted in the freshly dispersed VSMC cells from diabetic rats, the hyperglycemic condition had the greatest effect on the Ca^2+ ^signal from within the nuclear compartment. Using ratiometric Ca^2+^-sensitive fluorophores, the amplitude of the Ca^2+ ^signal to vasopressin stimulation (at time 0) in the nuclear compartment was shown to be decreased when A7r5 cells were grown in high glucose (Figure 4A, p < 0.0001). However, high glucose had no statistical effect on the Ca^2+ ^transient in the cytoplasm (Figure 4B). Likewise, the thapsigargin response was blunted in the nucleus in cells grown in high glucose (p < 0.001; Figures 4A and 4C) with little effect on the cytoplasmic response.

Under medium and low glucose conditions, the timing of the Ca^2+ ^transient was synchronous between the cytoplasmic and nuclear compartments of the A7r5 cells. Cells grown in medium and low glucose conditions (n = 74 and 97 cells, respectively) displayed 99% synchrony in the nuclear and cytoplasmic Ca^2+ ^signals produced by vasopressin or thapsigargin. Asynchrony between the nuclear and cytoplasmic Ca^2+ ^transients with stimulation was significantly increased in cells grown in high glucose, resulting in 13% of the total cells demonstrating asynchronous behavior (n = 138 cells).

**Figure 5:**
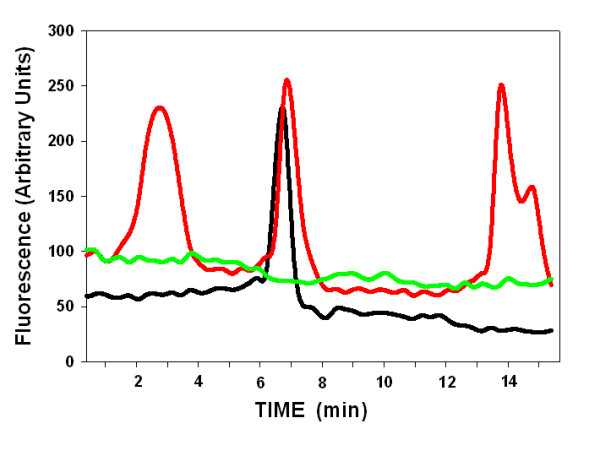
**High Amplitude Spontaneous Nuclear Ca^2+ ^Oscillations Occur in High Glucose**. In high glucose, A7r5 cells (n = 42) often demonstrated large spontaneous nuclear Ca^2+ ^transients. Spontaneous nuclear Ca^2+ ^activity in three individual cells, resting in high glucose conditions, depicts high amplitude oscillations in two cells represented by black and red lines (1 and 3 oscillations, respectively). In the same field, some of the cells failed to elicit any spontaneous oscillations as indicated with the green line.

In addition to asynchronous behavior in response to an agonist, 17% of the A7r5 cells exposed to high glucose demonstrated large magnitude spontaneous nuclear Ca^2+ ^transients well above 100% of baseline fluorescence levels in basal conditions (n = 42 cells; Figure 5). These high magnitude spontaneous oscillations appeared after 10 hours of incubation in high glucose media, but not before. In these cells, the cytoplasm demonstrated either a delayed oscillation or no oscillation at all (not shown). No spontaneous nuclear Ca^2+ ^oscillations were noted in the cells grown in medium (n = 61cells) or low (n = 34 cells) glucose.

### Diabetes and Hyperglycemia Altered Levels of SERCA Proteins

**Figure 6:**
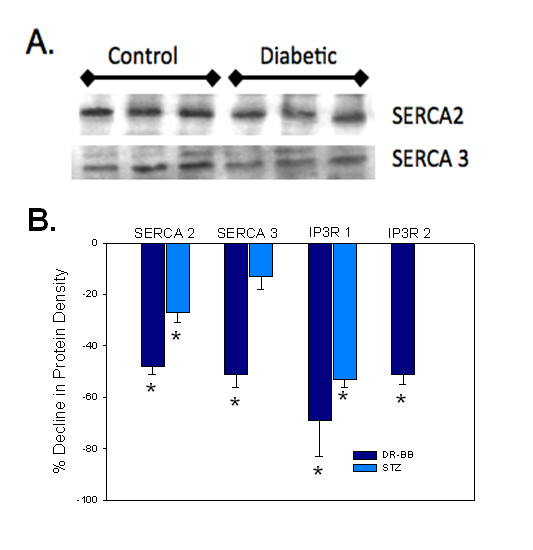
**Ca^2+ ^Regulatory Protein Levels Change with Diabetes**. A) Representative immunoblots for the SERCA proteins from control and diabetic DR-BB rat aorta. Each lane was loaded with protein from an individual animal. B) Plot illustrates the decline in aortic protein levels of the SR Ca^2+ ^ATPases (SERCA2 and SERCA3) and the IP_3 _receptors (type 1 and 2). Diabetes corresponded with a reduction in protein levels for all Ca^2+ ^regulatory proteins in both the STZ and DR-BB animal models, with one exception. The level of SERCA3 did not decline significantly with diabetes in the STZ model. * indicates a significant decline from non-diabetic rats, p < 0.05

In order to explain the dramatic changes in Ca^2+ ^signals caused by diabetes and hyperglycemia, intracellular Ca^2+ ^regulatory proteins including Ca^2+ ^pumps and channels were measured for protein levels and localization. Resting intracellular Ca^2+ ^levels in VSMCs are maintained by plasma membrane and intracellular Ca^2+ ^pumps (SERCA). The amount of the SR Ca^2+ ^pump (SERCA2 and SERCA3) protein in aortic VSMCs was significantly lower with diabetes in both animal models tested. Figure 6A illustrates a typical immunoblot from the aorta of DR-BB diabetic rats and their matched controls. Each lane represents the protein extract from a single animal. Levels of GADPH were not altered by diabetes in either animal model. Thus, GADPH was used to normalize the protein band density values, provided in Table 3. To illustrate the changes, the percent decline in the mean protein band density normalized per GAPDH content, in the aorta from diabetic DR-BB and STZ animals was compared to nondiabetic controls and plotted in Figure 6B. The figure shows that in the genetically-prone DR-BB animals there was a 48% decline in SERCA2 levels and a 52% decline in SERCA3 when compared to the aorta of matched non-diabetic rats. These declines were consistent when VSMCs from the femoral arteries were tested (Table 3). There was a 32% decline in the amount of SERCA2 from the femoral artery of STZ diabetic rats compared to the non-diabetic controls. Only SERCA3 levels in the STZ-induced diabetic rat aortas failed to demonstrate a significant difference when comparing the values from the diabetic to non-diabetic control animals.

**Table 3:** Levels of Ca^2+ ^Regulatory Proteins are Changed with Diabetes

	STZ-Induced Diabetic Rat Model				DR-BB Diabetic Rat Model	
**Protein Density** **(arbitrary units)**	**Control** **Aorta**	**Diabetic** **Aorta**	**Control Femoral**	**Diabetic Femoral**	**Control** **Aorta**	**Diabetic** **Aorta**

**SERCA 2**	165 ± 4	121 ± 12 *	120 ± 15	81 ± 9 *	108 ± 3	56 ± 2 *

**SERCA 3**	69 ± 19	60 ± 14	161 ± 13	110 ± 15 *	78 ± 5	38 ± 15 *

**IP_3 _R type 1**	40 ± 9	19 ± 8 *	213 ± 22	228 ± 28	135 ± 23	42 ± 6 *

**IP_3 _R type 2**	ND	ND	ND	ND	37 ± 4	18 ± 3 *

**RyR**	38 ± 5	95 ± 8 *	ND	ND	11 ± 5	72 ± 59

*Indicates a significantly different value when compared to respective controls (p < 0.05)

ND = not done

**Figure 7:**
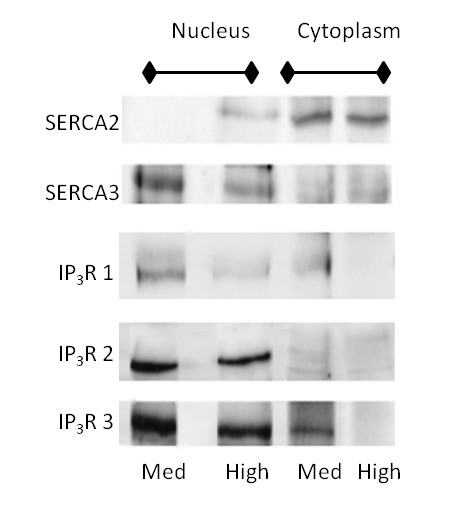
**Ca^2+ ^Regulatory Protein Levels Altered in Cultured VSMC Grown in High Glucose**. Immunoblots of the SERCA and IP_3_R isoforms in cytoplasmic and nuclear extracts from A7r5 cells grown in high or medium glucose concentrations are shown. SERCA2 protein levels remained steady in the cytoplasm, but an increase was observed in the nuclear extract from cells in high glucose. SERCA3 in the cytoplasm was detected at equally low levels in either glucose concentration. Nuclear SERCA3 levels were reduced with hyperglycemic treatment. All IP_3_Rs showed a reduction in protein levels in response to high glucose.

Figure 7 depicts the protein levels and compartment localization of different isoforms of Ca^2+ ^channels and pumps when A7r5 cells were grown in medium or high glucose conditions. In immunoblotting experiments, the amount of SERCA2 protein in the cytoplasmic fraction remained unchanged with high glucose, but SERCA2 levels increased in the nuclear extract with high glucose treatment, although it is important to note that the nuclear SERCA2 levels were still low with high glucose exposure. In low and medium glucose conditions, there was no detectible SERCA2 in the nuclear fraction, but in high glucose a clear band appeared. In contrast, SERCA3 was detected primarily in the nuclear extracts with cytoplasmic bands faintly visible. There was an average of a 60% decrease in the amount of SERCA3 in the nucleus with high glucose treatment. The majority of HSP-90 was found in the cytoplasm while USF-2 was strictly nuclear, validating the purity of the nuclear and cytoplasmic extracts (not shown).

### Diabetes and Hyperglycemia Alter Distribution of SERCA Proteins

**Figure 8:**
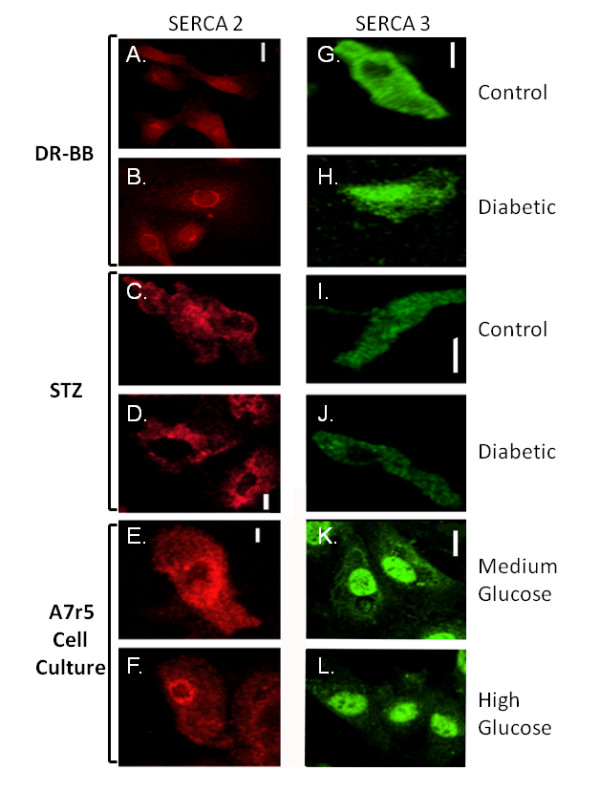
**SERCA2 and SERCA3 Shift to a More Nuclear and Perinuclear Distribution in Conditions of Diabetes and Hyperglycemia**. Immunofluorescence shows the distribution of proteins in cells from control and diabetic rats from both the DR-BB (A, B, G, and H) and STZ diabetic model (C, D, I, and J), and in A7r5 cells cultured with medium (E and K) or high glucose concentrations (F and L). Examples show a more localized perinuclear and nuclear distribution of SERCA2 in both animal models when diabetes was developed. In addition there was a decrease in the total fluorescence for SERCA3 in both animal models. Hyperglycemia also induced a redistribution of SERCA2 and SERCA3 to the nucleus.

While immunoblots using fractionated samples gave clues as to the distribution of the Ca^2+ ^regulating proteins, immunofluorescence provided more detailed information. SERCA2 immunostaining illustrated the intricate web of SR with a more pronounced perinuclear staining in cells from the DR-BB rats (Figure 8A). Diabetes induced a further shift in fluorescence to the nucleus in DR-BB rats with a decline in total cellular fluorescence intensity (Figure 8B). Interestingly, 6% of the cells from controls and 21% from diabetic DR-BB animals were positive for nuclear staining for SERCA2. The decline in total SERCA2 in the cells from diabetic animals was noted in both rat models (Figure 8A-D). In the A7r5 cultured VSMCs, SERCA2 had a more widespread distribution in cells grown in medium glucose (Figure 8E). Identical to the changes noted in the cells from the diabetic animals, there was a distinct localization to the nuclear SR with a decline in peripheral SR staining with high glucose treatment (Figure 8F). This finding is also consistent with the immunoblot results shown in Figure 7.

**Figure 9:**
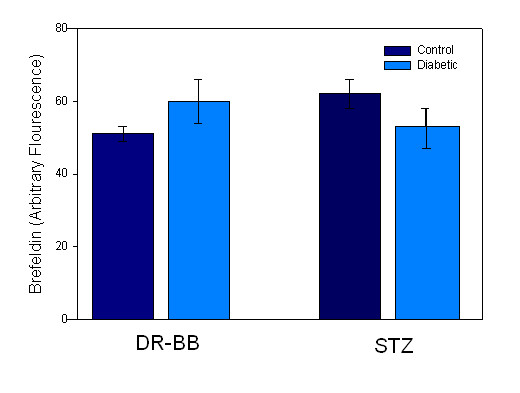
**Marker for SR Protein Levels Are Not Altered with Diabetes**. Fluorescence values from cells stained for SR area, using Brefeldin A as a marker, showed no differences between cells from control of diabetic animals, regardless of the type of diabetic animal model.

SERCA3 immunostaining patterns were generally the same in cells from both animal models exhibiting cytoplasmic staining with partial nuclear exclusion (Figure 8G-L). However, in 17% of the cells from either control or diabetic DR-BB rats, nuclear staining was noted (Figure 8H). Overall, diabetes had no significant effect on the location of the SERCA3 protein in aortic VSMCs. In cultured A7r5 cells, SERCA3 had strong nuclear staining with little cytoplasm staining, which was not altered in the high extracellular glucose condition (Figure 8K and 8L). In order to determine whether the distribution of the SR changed with high glucose, cells were double labeled with SERCA 2 and an SR stain, Brefeldin A. Brefeldin A levels were not affected by diabetes (Figure 9) or hyperglycemia when analyzed for cellular fluorescence values or for subcellular distribution (not shown).

### Diabetes and Hyperglycemia Altered Levels of Intracellular Ca^2+ ^Channels

The channels responsible for releasing Ca^2+ ^from the intracellular stores are the inositol trisphosphate receptor (IP_3_R) and the ryanodine receptor (RyR). There are three subtypes of IP_3_Rs, and each was examined in the aorta of the diabetic animal models and in cultured cells exposed to hyperglycemic conditions. Type 1 IP_3_Rs (IP_3_R-1) showed a significant decline in levels in the aortas from both models of diabetes with a 69% decline in the DR-BB diabetic animals and a 53% decline in the STZ rats (Figure 6B, Table 3). Type 2 IP_3_R (IP_3_R-2) were only tested in the genetically-prone DR-BB model of diabetes, showing a significant decline with diabetes. IP_3_R type 3 (IP_3_R-3) levels were not detected in amounts allowing immunoblot analysis of protein levels in either animal model.

A reduction in the levels of the IP_3_R prompted investigation of the other major SR Ca^2+ ^release channel in smooth muscle cells, the RyR to determine whether its subcellular distribution changed, possibly in response to changes in the IP_3_R. We hypothesized that the RyR could be either down-regulated due to a decline in cellular Ca^2+ ^signaling or upregulated as a compensatory mechanism for the decline in IP_3_R protein levels and cellular signaling. The RyR, increased dramatically with diabetes, regardless of the animal model (Table 3). In the STZ rats there was 2.5 times more RyR measured in the aorta when compared to non-diabetic controls. Interestingly, in the DR-BB rats there was an exquisite correlation between glucose levels and the amount of RyR. If the blood glucose was greater than 300 mg/dL on the day of sacrifice, then higher RyR levels were noted. However, if the diabetic rat had a lower glucose reading on that day, even if they had been hyperglycemic in the past, the changes in the RyR levels were minimal. This variability resulted in a lack of statistically significant differences found when comparing the diabetic to the control animals (Table 3).

In cultured VSMCs exposed to hyperglycemia, a decreased amount of protein for all three IP_3_R subtypes was observed under high glucose conditions (Figure 7). The levels of IP_3_R-1 decreased nearly 2.5 fold with high glucose exposure (Table 3). Although there was variability in the amount of protein normally present between the cytoplasm and nuclear compartments, in all cases high glucose treatment reduced the amount of IP_3_R-1 in each compartment. IP_3_R-2, detected mainly in nuclear extracts decreased 20% with high glucose exposure (Figure 7). IP_3_R-3 was detected in both the nucleus and cytoplasm in medium glucose conditions. However, in high glucose the levels decreased an average of 90% in the cytoplasm and decreased 30% in the nucleus (Figure 7).

### Diabetes and Hyperglycemia Altered the Distribution of Intracellular Ca^2+ ^Channels

**Figure 10:**
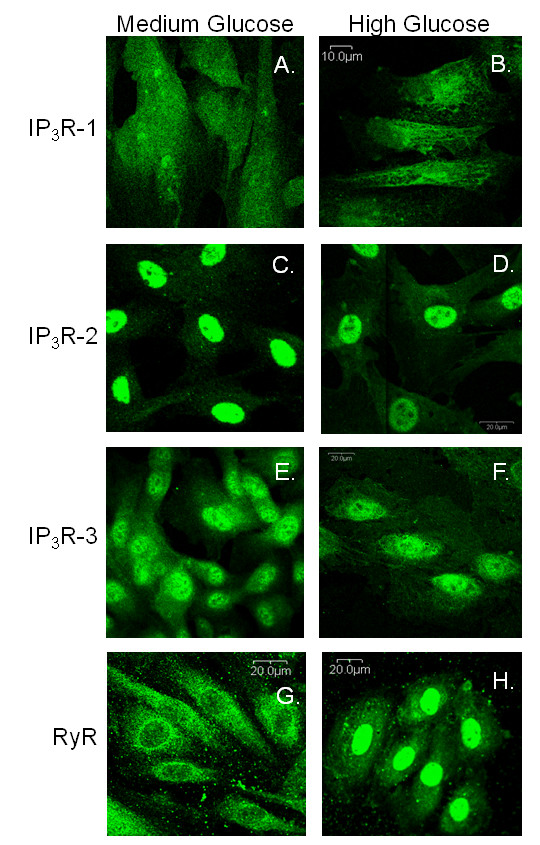
**IP_3_R and RyR Protein Distribution Changes with High Glucose Treatment**. Immunofluorescence experiments picture the relative location of each IP_3_R isoform in the cells grown in medium and high glucose. Few changes were noted in the distribution of IP_3_R-1 (A and B). There was a loss in fluorescence in IP_3_R-2 (C and D) and IP_3_R-3 (E and F) with high glucose treatment are shown. Cells grown in medium glucose conditions displayed primarily cytoplasmic staining of the RyR with perinuclear localization (G). RyR in cells grown in high glucose exhibited strong nuclear localization (H).

The IP_3_R-1 immunostaining displayed a generally even cellular distribution in cells from control and diabetic rats with a significant overall decline (p < 0.002) in cellular fluorescence intensity in the VSMCs from diabetic aortas (not shown) and from cultured A7r5 cells (Figure 10A and 10B). IP_3_R-2 labeling was less intense in the cells from both models of the diabetic rats compared to non-diabetic controls, but there was no change in the pattern of immunostaining for the IP_3_R-2. In contrast, IP_3_R-2 was localized strictly to the nuclear regions in both experimental conditions in A7r5 cells (Figure 10C and 10D). While IP_3_R-3 could not be detected sufficiently by immunoblot analysis, it could be evaluated by immunofluorescence. Like the other subtypes, IP_3_R-3 demonstrated a significant decrease in the total fluorescence with diabetes (in both animal models) and when cultured in hyperglycemia (p < 0.02; Figure 10E and 10F).

RyR immunostaining was primarily cytoplasmic with nuclear exclusion as the most common pattern, although some cells showed a more generalized staining in control that included the nuclear region. While the distribution of RyR was similar to the IP3R pattern, the fluorescence intensity was affected by diabetes in a manner that was just opposite to the IP3R response. Consistent with the protein levels measured with immunoblotting, the VSMCs from diabetic rats demonstrated significantly higher levels of RyR immunostaining compared to the cells from control animals (not shown). This was true of both diabetic animal models and A7r5 cells grown in high glucose concentrations. Immunostaining of the RyR demonstrated primarily patchy cytoplasmic staining with strong perinuclear localization (Figure 10G). A predominant relocation to the nucleus was observed for the RyR when cells were grown in high glucose (Figure 10H).

## Discussion

VSMC dysfunction and subsequent disease is the main cause of mortality in type 1 diabetes, but the underlying mechanisms are largely unknown. In normal (medium) glucose conditions reported here, the distribution of the IP_3_R subtypes, RyR, and SERCA were similar to other studies using primary aortic SMCs [23], rabbit aortic SMCs [24], and cultured cell lines [25]. Studies involving rat heart [26], islet cells and kidney [27] using diabetic animal models have demonstrated alterations in IP_3_R-1, SERCA2 and SERCA3 expression in a manner very consistent with our results from VSMCs.

The results of this study clearly demonstrate changes in the regulatory proteins associated with intracellular Ca^2+ ^storage and release in aortic and femoral VSMCs. These changes lead to altered Ca^2+ ^signaling in the cytoplasmic and nuclear compartments in response vasopressin. VSMCs from two different diabetic rat models showed a decline in the levels of IP_3_R and SERCA protein with diabetes. Changes in the distribution of the IP_3_R subtypes and SERCA subtypes were dependent on the rat model, but fully supported the immunoblot results of overall decreased amounts. To measure the relative role of hyperglycemia on the above mentioned changes, rat aortic cultured cells (A7r5) were exposed to various concentrations of glucose. Like the freshly dispersed cells from the diabetic animals, cultured cells exposed to high glucose had blunted Ca^2+ ^responses to vasopressin and decreased levels of Ca^2+ ^regulatory proteins. Thus, the changes noted in vSMCs from diabetic animals were likely due to exposure of the vSMCs to hyperglycemic conditions rather than adaptation of the cells to the complex disease of diabetes. This is the first study to report of both diminished levels and subcellular redistribution of key Ca^2+ ^regulatory proteins in cultured and freshly dispersed VSMCs.

Previous studies have described a blunted Ca^2+ ^response in VSMCs from diabetic animals [28-30]. Unique to this study was the identification of separate nuclear and cytoplasmic responses, and the presence of spontaneous nuclear Ca^2+ ^oscillations. Such oscillations in A7r5 cells have been reported to be associated with intracellular Ca^2+ ^stores and entry of Ca^2+ ^from the extracellular milieu [31,22]. Others have shown spontaneous Ca^2+ ^sparks occurring around the nucleus of primary VSMCs [32]. The effect of glucose on the spontaneous nuclear oscillations has not been previously published. In this study, the high magnitude spontaneous oscillations in resting cells bathed in high glucose were intracellular Ca^2+ ^release events as all experiments were performed in zero extracellular Ca^2+^.

In order to thoroughly examine the effects of hyperglycemia on VSMC function, two different animal models of type 1 diabetes were utilized; the toxin-induced STZ diabetic rat and the immune/inflammation model of type 1 diabetes, the DR-BB rat. The differences noted between the two models were minor, but in general the diabetes induced changes were more robust in the DR-BB rats. It is possible that any small differences we noted between rat models were a function of the rat strain, the method of diabetic induction, or the administration of insulin to the DR-BB animals.

In VSMCs, much of the ability to remove Ca^2+ ^from the cytoplasm is dependent on the SERCA proteins on the SR and nuclear envelope, estimated to be responsible for over 40% of the Ca^2+ ^buffering in VSMCs [33,34]. Importantly, by maintaining low intracellular Ca^2+^, SERCA inhibits migration of VSMCs into the intima of the vessel [35], a common hallmark of diabetes-induced vascular disease. In general, SERCA2 and SERCA3 protein levels were decreased and their distribution altered in the diabetic rats. Brefeldin A staining of the SR did not show a corresponding loss or reorganization of the SR with hyperglycemia, suggesting that these were pump-specific changes. In contrast to a previous report [36], we did not measure an increase in degradation in SERCA proteins with diabetes. In VSMCs from diabetic animals, SERCA2 shifted from a generalized SR distribution to a perinuclear location coupled with an overall decrease in the SERCA protein levels. SERCA2 is the primary Ca^2+^-ATPase of the SR in VSMCs and is thought to be most important in cytoplasmic Ca^2+ ^buffering [37]. Changes in the thapsigargin-induced Ca^2+ ^transient suggest that the decrease in protein levels affected the ability of the cells to buffer Ca^2+ ^via SERCA as has been shown in other labs [38].

The function of SERCA3 in VSMCs still remains elusive. Originally SERCA3 was reported to be absent from muscle cells. However, thorough examination of tissues with subtype-specific antibodies, revealed that at least one member of the SERCA3 family was equally expressed in all tissues tested [39]. In human vascular endothelial cells, chronic stimulation with histamine caused an upregulation in the SERCA3 protein, which allowed quicker sequestration of Ca^2+ ^from the cytoplasm following stimulation [40]. Given such results in endothelial cells, it is reasonable to consider that a decrease in the SERCA3 levels could partially explain the delayed and blunted response to thapsigargin in the VSMC shown here. The importance of this little defined protein should not be overlooked, as the SERCA3 locus has been implicated in the genetic susceptibility in human type 2 diabetes [41].

Vascular reactivity to vasopressin in diabetes varies between vascular beds [42,43]. For this reason, the protein levels and Ca^2+ ^responses were tested in VSMCs from both the aorta and the femoral arteries. The diabetes-induced changes in protein levels and in the Ca^2+ ^responses were the same in VSMCs from either location with the exception of SERCA2 levels, which were increased in the femoral artery, but decreased in the aortic VSMCs in the STZ rat. This increase in the femoral artery agrees with a study finding increased SERCA activity and levels in resistance arteries from diabetic dyslipidemic pigs [44]. SERCA2 may be of more functional importance in the resistance arteries so these findings may serve as a cautionary note for researchers drawing conclusions from aortic tissue alone.

When analyzing the Ca^2+ ^channel activity, the results presented here build on earlier work by Ma et al., illustrating a decline in IP_3 _responses in VSMCs from STZ treated rats [28]. The Ma paper showed a decline in vessel contractility and in intracellular Ca^2+ ^responses via IP_3 _receptors and Ca^2+ ^influx across the plasma membrane. A decrease in IP_3_R-1 levels in diabetic arteriolar SMCs has been published previously [45]. The current study focused the question on the level of specific Ca^2+ ^regulatory proteins on the SR by conducting live cell experiments in zero extracellular Ca^2+ ^conditions. Further, we showed that the findings were not dependent on the mode used to induce diabetes, using either a toxin-induced rat model or genetically-bred animals, as the results were generally consistent in the aorta and femoral arteries of the STZ and DR-BB animals. The results suggest that the decreased activity in the VSMCs associated with diabetes is due to shared changes in the SR Ca^2+ ^release channels (IP_3_R) and SERCA resulting in diminished Ca^2+ ^transients, especially in the nuclear compartment. In addition to selectively activating genes, nuclear Ca^2+ ^may also play an important role in stabilizing mRNA in a variety of cell types [46]. The functional impact on differential gene expression of decreased Ca^2+ ^responses in VSMCs as a result of diabetes is a question that should be intensely pursued, given the findings of this study.

The similarity of the results from identical experiments conducted in freshly dispersed cells from diabetic and control rats, and cultured rat aortic cells grown in normal and high glucose are striking and underlie the importance of chronic exposure to glucose as a major inducer of changes in intracellular Ca^2+ ^regulation. Previous studies have shown direct effects of glucose on cultured cells, but have typically worked with primary cultures [47,48,35]. In such cases 20-25 mM Ca^2+ ^in the extracellular media was sufficient to elicit a response similar to that seen in cells from diabetic animals. Due to the fact that we utilized a cultured cell line in which the normal growth media contained 25 mM glucose (referred to as medium in this study), we had to increase the glucose concentration dramatically to evoke a diabetes-like response. While the sensitivity to glucose was different in the culture cells, the other characteristics of the glucose-induced change in freshly dispersed and cultured cells were exceedingly similar.

The importance of separating the nuclear Ca^2+ ^response from cytoplasmic may not be immediately clear. We are not the first to report on selective Ca^2+ ^signals in the nuclear that were unique from the cytoplasm as Bkaily et al demonstrated in neurons [49,50]. Nuclear and cytosolic Ca^2+ ^have been reported to be regulated independently in several cell types [51,52]. Zones of Ca^2+ ^signaling around and within the nucleus of smooth muscle cells have led some to suggest that clusters of lysosomes with high densities of RyR form in the perinuclear region forming physical junctions with the SR to comprise a trigger zone of Ca^2+ ^[53]. The result of the depressed nuclear Ca^2+ ^signal with high glucose is unknown, but it is likely that VSMCs have nuclear-specific Ca^2+ ^transcription regulated pathways just as neurons have been shown to possess [54,55].

## Conclusion

While diabetes is a complex disease that includes hyperglycemia, hypoinsulinemia or hyperinsulinemia and numerous adaptations in metabolic proteins as well as changes in regulatory factors, it appears that high glucose is predominantly responsible for the decreased IP_3_-induced Ca^2+ ^transients noted previously in VSMCs from diabetic animals. This is likely due to the altered distribution and/or levels of Ca^2+ ^regulatory proteins including the IP_3_R, RyR, and SERCA. Determining how diabetes changes protein distribution and amounts is paramount, as these changes clearly have downstream effects within the cells that may be linked to cell proliferation and migration.

## Competing interests

The authors declare that they have no competing interests.

## Authors' contributions

YS carried out the confocal experiments and protein analysis as partial completion of her dissertation requirements. IS oversaw all immunoblot experiments and their analysis. RL was responsible for all animal work and participated in the design of the experiments using the DR-BB and STZ rats. LSB oversaw all work and wrote the manuscript. All authors read and approved the final version of the manuscript.
